# The New Physiology in Surgical and General Practice

**Published:** 1911-12

**Authors:** 


**The New Physiology in Surgical and General Practice.** By A. Rendle Short, M.D., B.S., B.Sc., F.R.C.S. Pp. vii., 201. Bristol: John Wright & Sons Ltd. 1911.

The author of this book is at once a surgeon and a physiologist. This dual capacity has permitted him to ascertain that the physiologist knows little of the triumphs of modern surgery, and that the surgeon is too busy to keep abreast with the advances of the "new physiology." Hence this successful and useful compilation. Our chief criticism is upon its brevity. There are many other subjects which might have been included, while an account—in simple language—of recent work in the domain of physical and chemical physiology would have been useful.

We have termed it a compilation, but this is not strictly true, for Dr. Rendle Short has included his recently-published work upon the action of cutaneous anæsthetics and some observations made by Dr. R. E. Thomas upon nutrient enemata.

The subjects discussed include the thyroid and parathyroid glands; the pituitary gland; studies in digestion and absorption; blood pressure; hemorrhagic diathesis; uric acid and other urinary deposits; acidosis and diabetes; chloroform poisoning; nerve injuries; spinal cord and cerebral localisation.

The text is easy to read, although a trifle "idiomatic" in places, and the index is well arranged and sufficiently full.

We welcome this book because of its practical aims and its innate usefulness in daily practice. It ought to be read by every practitioner. There is much in it also that stimulates thought and inspires inquiry. Whether the author is justified in his many suggestions time alone can tell.

At all events, he must have the credit of writing an intelligent review upon recent physiology and its possible practical applications.

The chapters on cerebral localisation and nerve injuries will be found most useful for purposes of reference.

